# Pyrazoles as potential anti-angiogenesis agents: a contemporary overview

**DOI:** 10.3389/fchem.2014.00078

**Published:** 2014-09-09

**Authors:** Konstantinos M. Kasiotis, Evangelia N. Tzanetou, Serkos A. Haroutounian

**Affiliations:** ^1^Laboratory of Pesticides Toxicology, Department of Pesticides Control and Phytopharmacy, Benaki Phytopathological InstituteAthens, Greece; ^2^Department of Animal Sciences and Aquaculture, Agricultural University of AthensAthens, Greece

**Keywords:** pyrazoles, synthesis, angiogenesis, cancer, VEGF, tyrosine kinase, inhibitors

## Abstract

Angiogenesis is a mulit-step process by which new blood vessels are formed from preexisting vasculature. It is a key rate limiting factor in tumor growth since new blood vessels are necessary to increase tumor size. In this context it has been shown that anti-angiogenic factors can be used in cancer therapy. Among the plethora of heterocyclic compounds administered as anti-angiogenesis agents, pyrazoles constitute one of the bottlenecks of this category. Currently, several pyrazole based compounds are administered or are in Phase II and III trials and new targets emerge. It is highly possible that the advent of the next two decades will lead to the discovery and use of additional pyrazoles whose anti-angiogenic profile will position them in the forefront of the battle of various malignancies. The present review is an attempt to focus on those pyrazoles that arise as anti-angiogenesis agents commenting both on the chemistry and bioactivity that these exhibit aiming to contribute to the perspectives that they hold for future research.

## Introduction

Azaheterocyclic compounds comprise a class of compounds that have demonstrated significant biological activities against various human diseases. Thus, their exploitation constitutes a constant goal of many medicinal chemistry groups implemented in the discovery of new lead pharmaceutical compounds. Angiogenesis—the formation of new blood vessels—is a multistep process that involves a complex interplay between a plethora of soluble factors, cell surface receptors, and extracellular matrix components (for first angiogenesis references see Algire and Chalkely, 1945; Folkman et al., 1971). Although angiogenesis is related to various physiological functions, an excessive or insufficient angiogenesis is connected with the appearance of diverse human diseases including retinopathy, rheumatoid arthritis, psoriasis, hemangioma, cancer (Folkman, 1995) and atherosclerosis (Sacar and Yaylali, 2011). For cancer particular, for which angiogenesis is regarded as a key stage, its inhibition constitutes an effective mechanism in slowing down tumor growth and malignancies.

The growth factors, angiogenic enzymes, endothelial specific receptors and the adhesion molecules which are involved in the expansion of vasa vasorum are all potential therapeutic targets. Amongst growth factors Vascular Endothelial Growth Factor (VEGF) is the major pro-angiogenesis factor, which is known to stimulate various steps of endothelial angiogenic activity, such as proliferation, migration, differentiation into vessel-like tubes (Leung et al., 1989). Its identification and prominent position in the angiogenic process has converted VEGF to an important therapeutic target. Fibroblast growth factors (FGFs) are heparin binding proteins that are also involved in the pathogenesis and subsequent progression of various cancer types such as endometrial cancer (Lee and Secord, 2014). In this view two other receptor tyrosine kinases and their ligands, namely angiopoietin/TIE-2 and ephrinB2/EphB4, contribute to later steps of vascular development like vessel branching and maturation.

Antiangiogenic agents—as stated by Ribatti (2014)—may be divided into two major categories: (a) indirect agents that block the activity of angiogenic molecules, or the expression of their receptors on endothelial cells, and (b) agents that directly affect endothelial cell function or survival.

Thus, the discovery of specific anti-angiogenesis agents constitutes an attractive therapeutic approach for the treatment of these diseases leading to the development of new highly active organic molecules. Therefore, the last decade several azaheterocyclic molecules have emerged as potent inhibitors of angiogenesis (see references in main text). The clinical research has demonstrated that research groups involved in anti-angiogenesis drugs for cancer are on a critical standpoint considering that these drugs' therapeutic activity in patients has been substantially lower than expected based on preclinical findings. In this context is essential to develop: (a) new compounds that will overcome the efficiency problem at clinical trials and (b) new assays to explore the dependency of individual tumors from any of these angiogenic pathways.

Among many azaheterocyclic structural entities, our group has devoted substantial effort to the preparation of pyrazole and isoxazole derivatives that might possess anti-angiogenic (Christodoulou et al., 2010; Tzanetou et al., 2012) and antiproliferative activity (Tzanetou et al., 2014). Our main goal was and still is to use commercially easily available molecules on which these azaheterocycles can be built upon and subsequently tested for their activity. Intrigued by our experience on the synthesis and bioactivity of such type of heterocycles, we herein present selected contemporary works on pyrazolic compounds that have demonstrated anti-angiogenic activity. In this view the reader is invited to revisit these research works and assess future perspectives of substituted pyrazoles in the anti-angiogenesis domain.

## Pyrazoles synthesis

The condensation of hydrazines with various 1,3-diketones constitutes the most common synthetic procedure for the construction of the pyrazole backbone, since initial methods not involving the 1,3-diketones require the adaptation of additional synthetic steps (Aggarwal et al., 2003; Bishop et al., 2004; Bhat et al., 2005). Other popular synthetic approaches are the: (a) 1,3-dipolar cycloaddition of diazo compounds with alkynes and (b) the reaction of α,β-unsaturated aldehydes and ketones with hydrazines. Pyrazole derivatives can also be prepared by the palladium-catalyzed four-component coupling of a terminal alkyne, hydrazine, carbon monoxide under ambient pressure and an aryl iodide as was recently reported by Ahmed et al. (2005). In 2012 the formation of substituted pyrazoles was reported by Panda and Jena (2012) using as substrates diarylhydrazones and vicinal diols, catalyzed by iron chloride. Xion et al. reported also the CuI-catalyzed coupling of *N*-acyl-*N*'-substituted hydrazines with aryl iodides affords *N*-acyl-*N′,N′*-disubstituted hydrazines regioselectively (Xiong et al., 2012). Pyrazolopyrimidnes'—an important class of pyrazole derived compounds with pronounced biological action—synthesis is vastly described in the bibliography and still attracts the interest of researchers (for a recent reference see Colombo et al., 2014). Additionally pyrazoles are synthesized by “elegant” approaches and incorporated in various fused core structures (Churruca et al., 2013), aiming to enhance their activity by modulating specific targets. In view of the importance of pyrazoles their synthesis has been efficiently reviewed by many research groups (Kumar and Jayaroopa, 2014); in the contemporary reviews apart from the synthetic pathways, medicinal chemistry groups describe the bioactive profile of the compounds (Chauhan and Kumar, 2013). Moreover Kumar et al. described with clarity natural products containing pyrazole moiety that have demonstrated significant bio-activity (Kumar et al., 2013b). The latter can be a key component in the efforts to explore and exploit chemical structures of bioactive natural products that might lead to compounds that will improve treatment or ideally cure patients.

### The herein reported pyrazoles

Pyrazolopyrimidines constitute the major pyrazole derivatives that have been investigated for anti-angiogenic action and selected works of the last 4 years are herein reported, though the medicinal attributes of this class was successively reviewed by Chauhan and Kumar (2013). In this context, emphasis is given to selected works and endeavors not reviewed until now. Most of pyrazolopyrimidines are kinase inhibitors which have been shown to play an important role in the inhibition of VEGF driven angiogenesis. Substituted, structurally flexible pyrazoles also possess a significant position amongst anti-angiogenic promising compounds. This category of compounds is exemplified by the current anti-angiogenic drug celecoxib, whose synthetic endeavors are presented below and reference to its bioactivity is also commented. Other pyrazole derivatives are also mentioned in this review article.

### Anti-cancer pyrazoles

Pyrazoles that exhibit anti-cancer activity have been reviewed by Chauhan et al. (2014). As correctly stated by the authors literature survey revealed that various N-substituted pyrazoles have been implemented as antileukemic, antitumor, antiproliferative, anti-angiogenic, DNA interacting, proapoptotic, autophagy, and antitubulin agents. Moreover these compounds are capable to exert remarkable anti-cancer effects through inhibition of different types of enzymes, proteins and receptors which play critical role in cell division. In this context the authors focused on the recent developments in pyrazoles along with their structure-activity relationship (SAR) that actually provides a good basis on how structures are related with binding to various receptors and how these compounds interplay with multiclass inhibitors. From this work it is evident that structural modifications of various pyrazoles have provided pharmacophore structures with medicinal interest and will probably continue to provide structural frameworks for further anticancer drugs development.

### Pyrazolopyrimidines

#### TAK-593

TAK-593 a pyrazolo carboxamide (substituted pyrazolo carboxamide), discovered and synthesized by a research group of a Japanese pharmaceutical company, is a highly potent VEGFR2 kinase inhibitor (Miyamoto et al., 2013). Molecular details for these imidazo[1,2-b]pyridazine derivatives are reported in this paper showing the crystal structure of a similar to TAK-593 derivative in complex with VEGFR2. This compound and TAK-593 differ in the substitution pattern of the central phenyl group (TAK-593 is methyl substituted at position 2 of the ring). Indicatively, the N1-nitrogen of the imidazo[1,2-b]pyridazine core interacts with the NH proton of Cys919; the CH proton at the 8-position of the core forms a CH·s O hydrogen bond with the carbonyl group of Glu917, while the cyclopropane moiety is suitably located in the solvent accessible region. The observation of a small hydrophobic pocket around the central phenyl ring, indicated the possibility of introducing a substituent on the phenyl moiety resulting in the synthesis of a series of substituted derivatives, including the bioactive molecule of TAK-593. Hence, from molecular docking standpoint the behavior of these compounds into the VEGFR2 is fully rationalized. Furthermore, its biochemical characterization was reported by Iwata et al. (2011). Kinase selectivity profiling revealed that this compound inhibited tyrosine kinases from the VEGFR and platelet-derived growth factor receptor (PDGFR) families. Two years later, the synthesis of this compound was published; it was based on previously reported work of this group on pyridazine molecules that also functioned as VEGFR2 kinase inhibitors (see Miyamoto et al., 2013 and references therein). Briefly, the preparation comprises of four synthetic steps involving as starting material an iodopyridazine. The major step of this pathway is the transformation of the latter to an imidazopyridazine. The end molecule displayed an IC_50_ value of 0.95 nM. Noteworthy it strongly suppressed proliferation of VEGF-stimulated human umbilical vein endothelial cells with a very low IC50 of 0.30 nM. The above findings postulate that TAK-593 is a lead compound and if systematically alter its structure—based on detailed docking study—different compounds can be yielded that are more effective and present possibly fewer side effects (for improvement of lead compounds see chapter 5 in Smith, 2013).

#### Pyrazolo[3,4-d] pyrimidines active on zebrafish model

**Figure 1:**
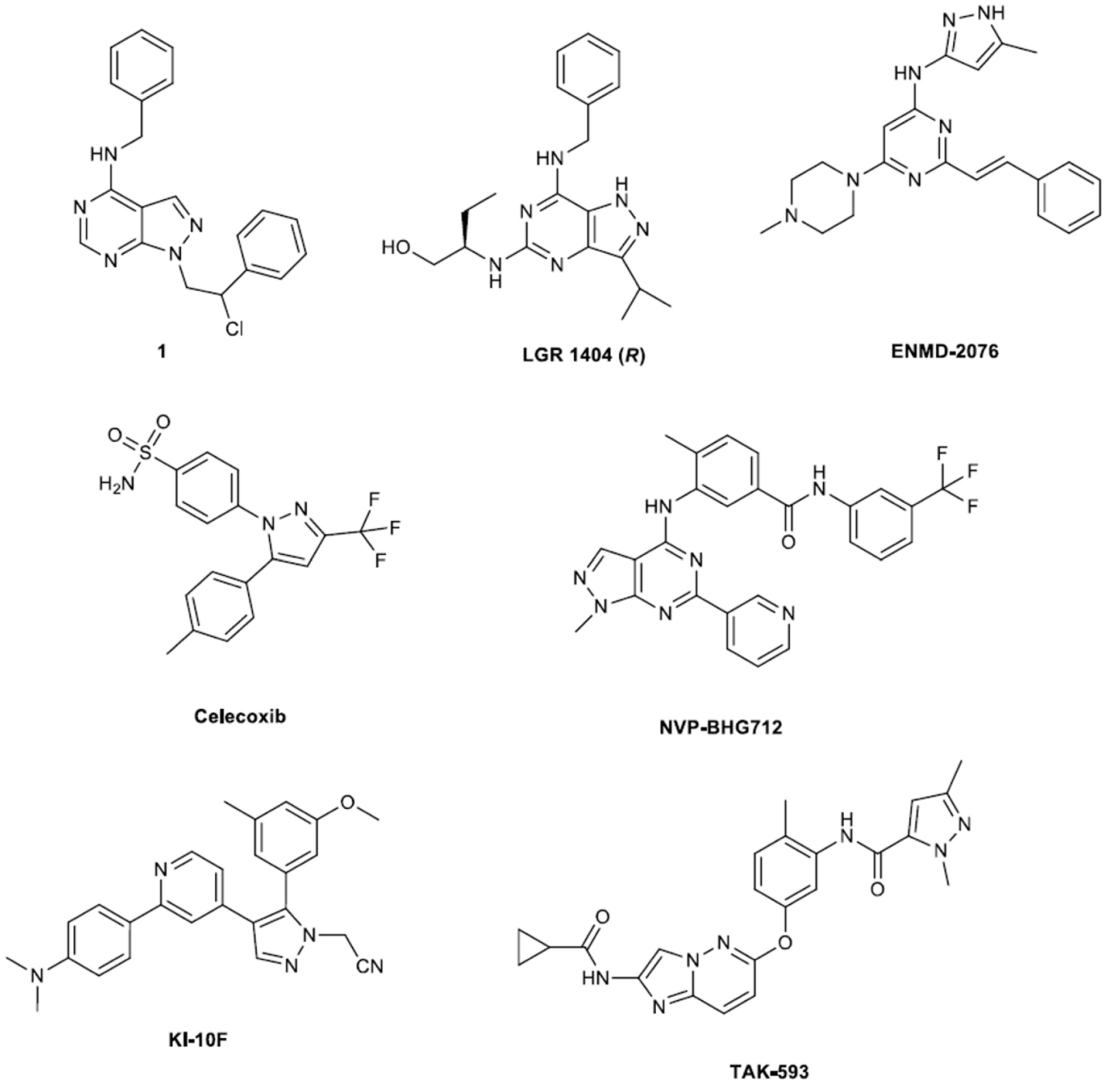
**Key structures of active anti-angiogenic pyrazoles**.

A combined targeted/phenotypic approach for the rapid identification of novel antiangiogenic compounds within *in vivo* efficacy was developed by Radi et al. (2012). Considering the important role played by the proto-oncogene tyrosine-protein kinase (c-Src) in the regulation of tumor angiogenesis, an in-house library of c-Src inhibitors was subjected to a sequential screening approach. Firstly, the authors applied a virtual docking and scoring procedure by submitting the VEGFR2 to a high throughput docking protocol (*in silico* screening on VEGFR2) so as to sufficiently select molecules for screening, considering that this approach is more robust the than pharmacophore based methods. The best compounds—which belonged to the pyrazolopyrimidines class (e.g., see compound 1, Figure 1)—were then subjected in *in vitro* screening on HUVEC cells, ADME profiling, formulation and *in vivo* testing on a zebrafish model. Thus, a promising antiangiogenic candidate, compound 1, able to interfere with the vascular growth of a zebrafish model at low micromolar concentration was identified.

#### An urea pyrazolo[3,4-d]pyrimidine

Many research groups try to “invest” on currently active compounds in order to prepare derivatives with enhanced activity. In a paper published in 2013 the structural optimization of a hit compound, 1-(4-(1H-pyrazolo[3,4-d]pyrimidin-4-ylamino)-phenyl)-3-(3-methoxyphenyl)urea, which exhibited inhibitory activity but low potency against FMS-like tyrosine kinase 3 (FLT3) and VEGFR2, was described (Yang et al., 2013). The authors developed a series of pyrazolo[3,4-d]pyrimidine derivatives based on structural modifications of specific atoms or subgroups, assisted by structure-activity relationship (SAR) analysis using cell—and transgenic-zebrafish-based assays. All of the target compounds were prepared from the general intermediate 4-chloro-1H-pyrazolo[3,4-d]pyrimidine, which was obtained through reaction of phosphorus oxychloride with a commercially available pyrazolopyrimidinone. The latter was reacted with various chlorine substituents whose synthesis is also reported in distinct schemes. These efforts led to the discovery of a number of compounds that exhibited both high potency against FLT3-driven human acute myeloid leukemia (AML) MV4-11 cells and a considerable anti-angiogenic effect in transgenic-zebrafish-based assays. One pyrazolo urea derivative, which exhibited the highest activity in preliminary *in vivo* anti-AML assays, was chosen for further anti-AML studies. The studies revealed that this compound can serve as multikinase inhibitor that potently inhibits FLT3 and VEGFR2. In an MV4-11 xenograft mouse model, a once-daily dose of compound 33 at 10 mg/kg for 18 days led to complete tumor regression without obvious toxicity. From bioactivity point of view, it is evident that the presence of an oxygen atom as a linker favors bioactivity and should be regarded in further synthetic efforts (suggesting that the phenoxy group should be maintained). Moreover substitution of the N-1 position is not beneficial for the activity, while discussion on the role of bridge groups and ring B moiety can help as well in the design of new molecules.

#### Trisubstituted pyrazolo[4,3-d]pyrimidines

In the frames of exploiting small molecular inhibitors of tyrosine kinase receptors Weitensteiner et al. have developed active trisubstituted pyrazolo[4,3-d]pyrimidines as novel anti-angiogenic compounds (Weitensteiner et al., 2013). Their synthesis was based on a condensation step of a methylsulfone compound with the respective amine. All seven tested compounds inhibited endothelial cell proliferation with IC_50_ values ranging from 1 to 18 μM [compound LGR-1404(R) is depicted in Figure 1]. The latter was not attributed to cytotoxicity, since none of them showed acute cytotoxic effects on endothelial cells at a concentration of 10 μM. The three most potent compounds also inhibited cell migration, chemotaxis and tube formation. Apart from the efficiency in *in vitro* testing the antiangiogenic potency of these compounds was tested with the *in vivo* chorioallantoic membrane (CAM) assays. The three compounds completely eliminated VEGF induced vessel formation. Again, structural modifications can provide improved compounds conditioning that appropriate molecular modeling study accompanies the synthesis. Specifically, targeted elongation of the side chain bearing the alcohol group or alkyl substitution of the amines can function as synthetic alternatives to the pursuit of LGR-1404 analogs.

### Small kinase inhibitor

Novartis group developed novel small molecular weight kinase inhibitors (Martiny-Baron et al., 2010). Amongst them NVP-BHG712 (see structure in Figure 1) inhibited erythropoietin-producing hepatocellular carcinoma, EphB4 kinase activity in the low nanomolar range, showing—in cellular assays—high selectivity for targeting the EphB4 kinase when profiles against other kinases in biochemical as well in cell based assays. Design of NVP-BHG712 was reinforced by molecular modeling of the kinase domain of EphB4 and further optimized using structure-activity-relationship data based on inhibition of EphB4 autophosphorylation in a cellular assay. Its synthesis has been described in a patent (see pertinent reference into the document). At *in vivo* level this molecule inhibits VEGF driven vessel formation, while it has only little effects on VEGF receptor activity *in vitro* or in cellular assays. From the above is evident that further exploitation of this structure should and will probably follow.

### Celecoxib

Celecoxib is a tricyclic compound that encompasses a pyrazole ring (see Figure 1). This compound acts as an excellent selective non-steroidal anti-inflammatory agent, which inhibits the enzyme cyclooxygenase-2 (COX-2). Its synthesis and developments of it, have been efficiently reviewed by Kumar et al., demonstrating that the main synthetic step is the condensation of 1,3-diketones with hydrazines (Kumar et al., 2013a). In this review improvements on the “classical condensation reaction” as well as development of prodrugs and derivatives of celecoxib are also presented.

The association of celecoxib with angiogenesis has been reported by many groups. In this context Wei et al. reported in 2004 that celecoxib inhibits VEGF expression and reduces angiogenesis and metastasis of human pancreatic cancer via suppression of Sp1 transcription factor activity (Wei et al., 2004). Additionally, and in contrast to its previously mentioned anti-angiogenic profile, researchers have shown that celecoxib can induce VEGF expression and tumor angiogenesis (Xu et al., 2011).

### Pyrazolobenzodiazepines

**Figure 2:**
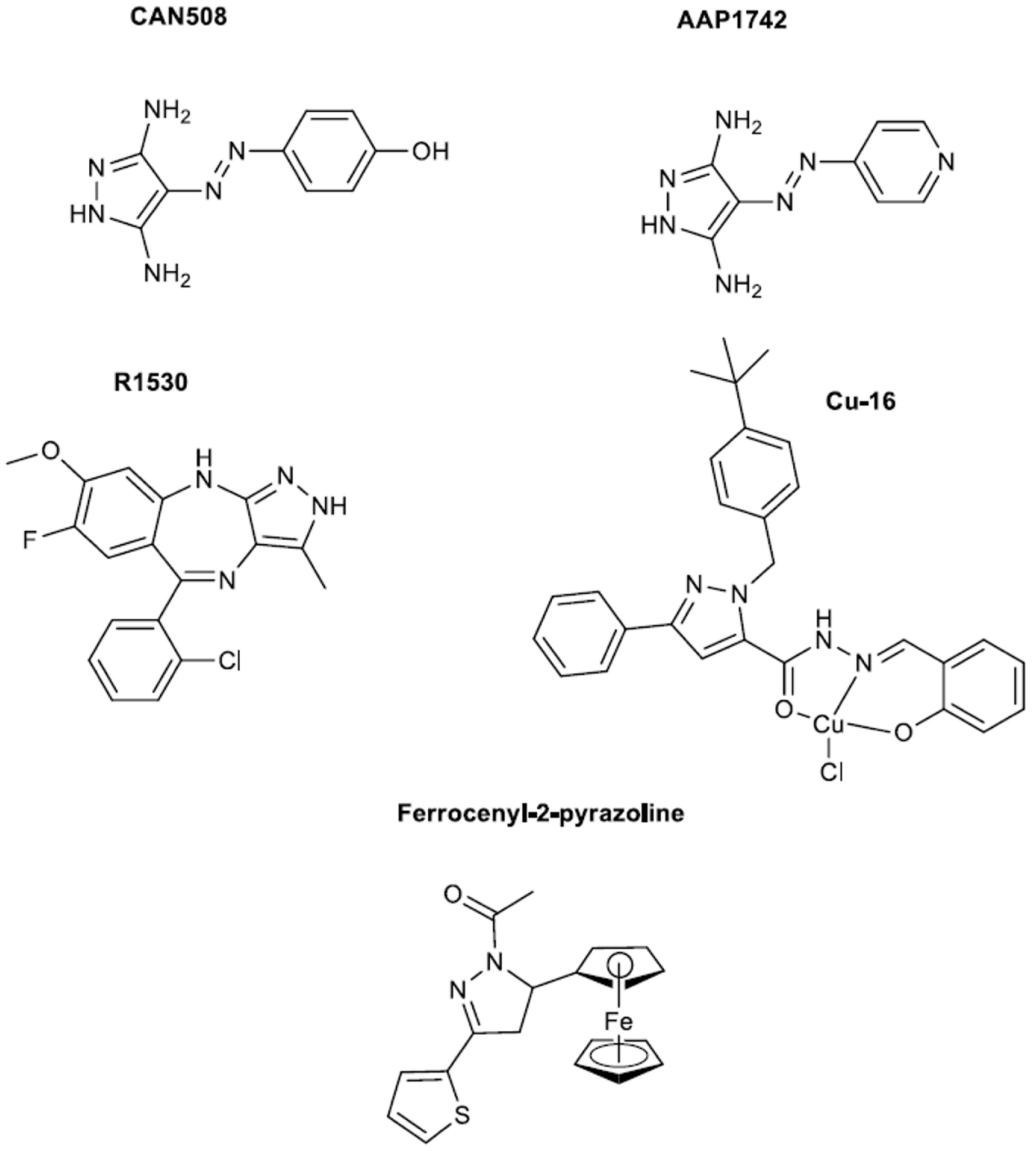
**Diamino pyrazoles, a pyrazolobenzodiazepine, copper and ferrocenyl complexes**.

In 2011 the preclinical evaluation of a novel multi-targeted agent R1530 (see structure in Figure 2) was reported by a group of Hoffman-La Roche (Kolinsky et al., 2011). The anti-proliferative activity of this compound was tested in a range of human tumor, endothelial and fibroblast cell lines. R1530 exhibited activity against tumor models *in vitro* and *in vivo* leading to the efficient angiogenesis inhibition. The synthesis was rationalized on the di-substitution at 7,8 positions of the fused benzene rings aiming to improve on the kinase inhibitory activities since an amine derivative previously synthesized (Liu et al., 2010) exhibited high kinase inhibitory activity however its ADMET (absorption, distribution, metabolism, and excretion) profile discouraged the human evaluation. The crystal structure of one analog with cyclin-dependent kinase 2 (CDK2) receptor as reported by Liu et al., showed that pyrazolobenzodiazepine core occupies the same site as the adenosine of ATP, and makes three critical H-bonds to the hinge region. Extensive discussion on the receptor binding sites is given in that paper. Later in 2013 same group reported the synthesis of R1530 and precursor of it, while they explained the rationale behind synthesis and structural modifications that took place (Liu et al., 2013). Synthesis was based initially on a condensation of a substituted aniline with a benzonitrile. The imine intermediate was transformed to a corresponding diazepinone and with a three step synthesis to the final pyrazolobenzodiazepine. In parallel the authors reported a convergent synthesis of R1530. Finally in that work R1530 as compared to a monosubstituted counterpart showed stronger inhibitory activities against angiogenesis-related receptor tyrosine kinases, indicating the feasibility of improving potency and selectivity for the angiogenesis-related kinases.

### Copper complex of salicylaldehyde pyrazole

A work published in 2010 was focused on complexes of copper and salicylaldehyde pyrazole hydrazine, which belong to the family of trisubstituted pyrazoles (see structure of Cu-16 complex in Figure 2) (Fan et al., 2010). The latter was an outcome of the increased anti-cancer activities of copper complexes (Brewer, 2001) and the anti-proliferative profile of previously synthesized pyrazoles (Chen et al., 2008; Xia et al., 2008). Syntheses of these complexes were based on published procedures before 2010 (Xia et al., 2008; Fan et al., 2009). From compounds synthesized the Cu-16 complex bearing a *tert*-butyl moiety promoted apoptosis in H322 cells through elevating the protein level of integrin β4.

### KI-10F an anticancer, anti-angiogenesis agent

A novel pyrazole derivative KI-10F [2-(4-(2-(4-(Dimethyl-amino)phenyl)pyridin-4-yl)-5-(3-methoxy-5-methylphenyl)-1H-pyrazol-1-yl), structure in Figure 1] acetonitrile was developed and evaluated in human colon cancer cells (Hong et al., 2012). Its synthesis is not described (only the formation of the HCl salt) into the paper however the specific compound inhibited angiogenesis both *in vivo* and *in vitro*, categorizing it as a chemotherapeutic candidate. Specifically KI-10F strongly suppressed the growth of human colon cancer cells and induced apoptosis by increasing the proportion of sub-G1 presenting apoptotic cells as well as causing cycle arrest at the G2/M phase. Moreover it decreased expression of HIF-1α and VEGF, and the HUVEC tube formation and migration, thus it inhibited the angiogenesis process.

### ENMD-2076

In the frame of constantly growing number of small molecules and antibody kinase inhibitors that have entered the global market for the battle of various cancer forms a research group published a preclinical development of a new azaheterocycle ENMD-2076 (see Figure 1) with pronounced antiangiogenesis and antiproliferative action (Fletcher et al., 2011). In this regard, the authors proceeded to the synthesis of ENMD-2076 according to patented procedure (PATENT-1^1^) and then assessed its biological activity. ENMD-2076 is a well-tolerated, orally active multi-target kinase inhibitor with a unique antiangiogenic/antiproliferative profile and provides strong preclinical support for use as a therapeutic for human cancers (Diamond et al., 2013; Matulonis et al., 2013).

### Diaminopyrazoles

CAN508 is a 3,5-diamino pyrazole (see Figure 2) that has been developed as an anticancer drug but has also been shown to inhibit angiogenesis (Krystof et al., 2011). This molecule represents a novel approach on anti-angiogenesis drug candidates which focuses on small simplified pyrazoles. The latter might open new frontiers on the efficient preparation of a plethora of small substituted pyrazoles. CAN508 synthesis has been reported in a patent and it also appears in a publication of Krystof et al. (2006). Briefly, it starts with a diazotization of arylamines and finalizes with a cyclocondensation of these hydrazones with hydrazines to furnish the desired compounds. CAN508 inhibited endothelial cell migration and tube formation. Its high selectivity toward the positive transcription elongation factor b (P-TEFb), suggested that P-TEFb may be a possible target for anti-angiogenic therapy. The same group in 2014 published a work on a novel member of this class of compounds; AAP1742 (Figure 2) was found to inhibit cyclic-dependent kinases 9 (CDK9) and reduced the viability of multiple myeloma cell lines in low micromolar concentrations (Jorda et al., 2014).

### Ferrocenyl pyrazolines

Recently novel ferrocene-containing N-acetylated-2-pyrazoline compounds were studied for *in vitro* inhibition of angiogenesis and human lung cancer growth (Bostancioglu et al., 2013). The compounds of interest were synthesized based on bibliographic procedures (see Bostancioglu et al., 2013 and references therein). The final tested products were prepared by reaction of intermediate chalcones with hydrazine hydrate to afford N-acetylated products. For testing two cell lines were used, a human non-small-cell lung cancer cell line (A549) and a human umbilical vein endothelial cell line. The cytotoxic activity was assessed by the methyl thiazol tetrazolium assay (MTT), apoptotic by 4,6-diamidinophenylindole and F-actin staining, antitumoral by colony forming ability assay and antiangiogenic activities by tube formation. The dose dependent manner of compounds activities was revealed by the assays with N-acetyl-3-(2-thienyl)-5-ferrocenyl-2-pyrazoline being the most potent in inhibition of capillary vessel formation (see structure in Figure 2). The latter can be used in the development of therapeutic agents for angiogenic-related diseases and cancer.

## Concluding remarks

Pyrazole molecules are in the forefront of organic chemistry due to their capacity to encompass various substituents which in sequence administer—to the synthesized molecules—unique medicinal properties. In this regard the interest on their chemistry will remain and possibly increase due to the need of more bioactive heterocylcles. As regards their angiogenesis implication it is a logical outcome after their initial implication in the anti-cancer drug portfolio which embraces angiogenesis. In that frame researchers have to concentrate on: (a) already reported anti-angiogenic pyrazoles, such as TAK-593 which exhibited activity at the nanomolar range, and pursue structural improvements that will lead to molecules with enhanced bioactivity and will be devoid of toxic side effects (b) explore pyrazolic compounds that have been developed to target other diseases but might possess anti-angiogenic action as well and (c) develop new pyrazole derivatives that will target the angiogenic cascade, incorporating novel or existing natural products' derived heterocycles.

### Conflict of interest statement

The authors declare that the research was conducted in the absence of any commercial or financial relationships that could be construed as a potential conflict of interest.

## Footnotes

^1^http://patents.justia.com/patent/7563787
